# Stimulation of MAP kinase pathways after maternal IL-1β exposure induces fetal lung fluid absorption in guinea pigs

**DOI:** 10.1186/1465-9921-8-27

**Published:** 2007-03-26

**Authors:** Reshma Bhattacharjee, Tianbo Li, Shyny Koshy, LaMonta L Beard, Kapil Sharma, Ethan P Carter, Chrystelle Garat, Hans G Folkesson

**Affiliations:** 1 Department of Physiology and Pharmacology, Northeastern Ohio Universities College of Medicine, Rootstown, OH 44272-0095, USA; 2 S/M Cardiovascular Pulmonary Research, University of Colorado Health Science Center, Denver, CO 80262, USA

## Abstract

**Background:**

We tested the hypothesis that maternal interleukin-1β (IL-1β) pretreatment and induction of fetal cortisol synthesis activates MAP kinases and thereby affects lung fluid absorption in preterm guinea pigs.

**Methods:**

IL-1β was administered subcutaneously daily to timed-pregnant guinea pigs for three days. Fetuses were obtained by abdominal hysterotomy and instilled with isosmolar 5% albumin into the lungs and lung fluid movement was measured over 1 h by mass balance. MAP kinase expression was measured by western blot.

**Results:**

Lung fluid absorption was induced at 61 days (D) gestation and stimulated at 68D gestation by IL-1β. Maternal IL-1β pretreatment upregulated ERK and upstream MEK expression at both 61 and 68D gestation, albeit being much more pronounced at 61D gestation. U0126 instillation completely blocked IL-1β-induced lung fluid absorption as well as IL-1β-induced/stimulated ERK expression. Cortisol synthesis inhibition by metyrapone attenuated ERK expression and lung fluid absorption in IL-1β-pretreated fetal lungs. JNK expression after maternal IL-1β pretreatment remained unaffected at either gestation age.

**Conclusion:**

These data implicate the ERK MAP kinase pathway as being important for IL-1β induction/stimulation of lung fluid absorption in fetal guinea pigs.

## Background

Experimental and clinical evidence support the notion that prenatal lung fluid absorption is critical for normal pulmonary gas exchange at birth. Albeit that some fluid may be expelled through the trachea and mouth during parturition [1], the majority is absorbed by lung epithelia secondary to active Na absorption [2]. Rising endogenous epinephrine levels near term contribute to a decreased alveolar fluid volume, increased Na absorption, and induction of lung fluid absorption [3-6]. Na absorption is driven by basolateral Na,K-ATPases [7] and apical epithelial Na channels (ENaC) [8-10] in the lung epithelial cells.

Cytokines, such as IL-1, have been proposed to signal parturition onset [11]. During a normal pregnancy, low amniotic IL-1β levels are present, but higher IL-1β levels are seen in preterm labor [12]. Many preterm infants suffer from fetal infection and/or the respiratory distress syndrome (RDS). It has been proposed that alveolar epithelial ion transport abnormalities may be important in RDS [13]. Experimental studies have suggested that cytokines could signal lung maturation [3,14-16]. Bry and colleagues [14] reported increased surfactant protein mRNA expression and improved lung compliance after intra-amniotic IL-1α administration. Maternal IL-1β exposure in guinea pigs induced fetal lung fluid absorption by activating the hypothalamus-pituitary-adrenal gland axis [3,16]. This led to fetal cortisol synthesis, which in turn increased membrane expression of β-adrenoceptors (βAR), Na,K-ATPases, and ENaC as well as induced fetal lung fluid absorption.

It has been proposed that mitogen activated protein kinases (MAP kinases) may regulate βAR stimulation of lung fluid absorption by affecting Na,K-ATPase membrane expression [17]. We thus decided to study if maternal IL-1β pretreatment activated MAP kinase pathways in fetal guinea pig lungs and if this would affect induction and stimulation of lung fluid absorption. Our hypothesis was that maternal IL-1β pretreatment induced MAP kinase signaling via cortisol synthesis/release. Consequently, the first aim of these studies was to measure MEK and ERK activation as pMEK and pERK expression in guinea pig fetal lungs at gestation days (D) 61 and 68 (term = 69D gestation). The second aim was to determine the MAP kinase pathway specificity of this response by measuring JNK phosphorylation. We could not measure p38 activation due to a lack of decent cross-reacting antibodies for guinea pigs. Finally, in order to functionally test the hypothesis, the third aim was to study if the MEK inhibitor U0126 affected lung fluid absorption when administered directly to the fetal lungs. We also examined the lungs for ERK expression after U0126 instillation. Since cortisol synthesis has been demonstrated as important for IL-1β induction of lung fluid absorption [3,16], the fourth aim was to study the effect of cortisol synthesis inhibition by metyrapone (MP) on ERK expression.

## Materials and methods

### Animals

Preterm Dunkin-Hartley guinea pigs (Hilltop Lab Animals, Inc., Scottdale, PA) were used (*N *= 123 divided on 31 litters). The timed pregnant guinea pigs were maintained at 12:12-h day-night rhythm and had free access to food (Standard guinea pig chow; Purina; Copley Feed, Copley, OH) and tap water. The Institutional Animal Care and Use Committee (IACUC) at the Northeastern Ohio Universities College of Medicine approved this study.

### Solutions and chemicals

A 5% albumin instillation solution was prepared by dissolving 50 mg/ml bovine serum albumin (BSA; Calbiochem-Novabiochem Co., La Jolla, CA) in 0.9% NaCl. In some studies, the MEK inhibitor U0126 (Cell Signaling Technology™, Beverly, MA) was added to the instilled fluid at a concentration of 10^-6 ^M.

The IL-1β pretreatment solution was prepared by dissolving 10 μg rat recombinant IL-1β (Sigma, St. Louis, MO) in 0.1% BSA in 0.9% NaCl. The dissolved IL-1β was aliquoted into vials containing 500 ng and stored frozen at -20°C until used.

The 11-β-hydroxylase inhibitor, metyrapone (MP; 2-methyl-1,2-di-3-pyridyl-1-propanone; Sigma), pretreatment injection solution was prepared by dissolving 62.5 mg/ml MP in 24% ethanol in 0.9% NaCl. Injection of 24% ethanol in 0.9% NaCl has earlier been demonstrated to be without effect on lung fluid absorption in guinea pigs [18].

### Pretreatments

Guinea pigs of 59 and 66D gestation were injected subcutaneously on the dorsal neck once daily with 250 ng/kg body wt IL-1β for 3 days. Control timed-pregnant guinea pigs were given injections of 0.9% NaCl at the same times. Lung fluid absorption studies were carried out on the morning of the last pretreatment day.

MP pretreatment was carried out over three days simultaneously with the IL-1β pretreatment. Subcutaneous MP injections were given twice daily (25 mg/kg body wt to reach a total daily dose of 50 mg/kg body wt) to guinea pigs of 59 and 66D gestation. In the morning of the day of the lung fluid absorption study, one half the daily dose was given. The MP dose was adopted from its higher ranges of clinical dosage.

### Surgery

Timed-pregnant guinea pigs were anesthetized by intraperitoneal (i.p.) injections of pentobarbital sodium (120 mg/kg body wt; Abbott Laboratories, Chicago, IL) and euthanized by intracardiac injections of 60 mg pentobarbital sodium. A laparotomy was rapidly done and the fetuses were carefully delivered. The umbilical cord was ligated to prevent bleeding. The fetuses were immediately euthanized by i.p. sodium pentobarbital (12 mg) mixed with 500 IU heparin (Elkins-Sinn, Cherry Hill, NJ). After euthanasia, an endotracheal tube (PE-190; Clay Adams, BD, Parsippany, NJ) was inserted via a tracheostomy. The fetuses were connected to a constant O_2 _flow (FIO_2 _= 1.0; Praxair, Akron, OH) and the lungs were expanded by adjusting the O_2 _flow to a constant positive airway pressure (CPAP) of 5 cm H_2_O. Fetuses were placed between heating pads to maintain body temperature during the studies. A temperature probe measured body temperature and heating was adjusted to maintain the temperature at 37–38°C. Airway pressure was continuously monitored by calibrated pressure transducers and analogue-to-digital converters and amplifiers (ADInstruments, Grand Junction, CO).

### Lung fluid absorption

Lung fluid absorption was studied as before [3,16]. Briefly, the albumin solution (10 ml/kg body wt) was instilled into the lungs through the endotracheal tube. Fetuses were briefly disconnected from the CPAP and the lungs were deflated by gently aspirating residual air with the instillation syringe. The instillation solution was instilled and withdrawn. This procedure was repeated four times to allow thorough mixing of instillate and pre-existing fetal lung fluid and the fluid was finally instilled. The fetuses were reconnected to the CPAP and remained on CPAP for 1 h. A 0.1-ml sample of instillation solution – lung fluid mixture (initial solution) was retained for protein measurement. After 1 h, remaining lung fluid was collected. Instillate, initial, and final solution protein concentrations were determined spectrophotometrically (Labsystems Multiscan Microplate Reader, Labsystems, Helsinki, Finland) by the Lowry method [19] adapted for microtiter plates.

Lung fluid absorption in ventilated [4,18], earlier *in situ *CPAP animals [3,5,16], and in our *in situ *CPAP animals was not significantly different. Moreover, in our recent study [3] we demonstrated that IL-1β injections did not cause significant intrauterine or fetal infection, nor did it affect the pulmonary endothelial or epithelial protein permeabilities.

### Specific protocols

Guinea pig fetuses of 61 and 68D gestation post conception were studied. Day of conception was set to the day when the timed-pregnant guinea pigs gave birth to their earlier litter, since guinea pigs enter estrus immediately after birth. All groups contained fetuses from at least two litters and all fetuses were studied for 1 h after fluid instillation.

#### Control

Preterm 61 (Control: *N *= 9; U0126: *N *= 7) and 68D (Control: *N *= 15; U0126: *N *= 10) gestation fetuses were delivered by abdominal hysterotomy from 0.9% NaCl injected timed-pregnant guinea pigs. The 5% albumin solution with and without the MEK inhibitor, U0126 (10^-6 ^M), was instilled.

#### IL-1β

Preterm 61 (Control: *N *= 10; U0126: *N *= 10) and 68D (Control: *N *= 11; U0126: *N *= 11) gestation fetuses were delivered by abdominal hysterotomy from IL-1β-pretreated timed-pregnant guinea pigs. The 5% albumin solution with and without U0126 was instilled.

#### Cortisol inhibition

Preterm 61 (Control: *N *= 6; IL-1β: *N *= 6) and 68D (Control: *N *= 11; IL-1β: *N *= 6) gestation fetuses with or without IL-1β pretreatment of were delivered by abdominal hysterotomy from MP-pretreated timed-pregnant guinea pigs. The 5% albumin solution was instilled.

### Western blot protocols

Lung tissue was obtained from four fetuses in each group above after the 1-h lung fluid absorption study. The lung tissue was homogenized in T-Per™ Reagent (Pierce, Rockford, IL) containing protease inhibitors (aprotinin; 30 μg/ml; leupeptin; 1 μg/ml; Sigma) on ice. The tissue homogenate was centrifuged at 10,000 *g *(5 min, +4°C). The supernatant (membrane fraction) was collected and aliquoted in multiple vials for each sample and snap-frozen in liquid nitrogen unless the western blot was carried out on the same day. One vial was used for determining sample protein concentration to ensure equal loading of the electrophoresis gel. Aliquots were stored at -80°C until analysis.

Polyacrylamide gel electrophoresis and transfer to nitrocellulose membrane (Pierce) were carried out using standard protocols. After electrophoresis and transfer, the nitrocellulose membrane was placed in blocking buffer (SuperBlock™ Dry Blend blocking buffer in tris buffered saline (TBS); Pierce) for 1 h.

#### MAP kinase pathway

Anti-pMEK, MEK, pERK, ERK, and pJNK antibodies were obtained from Cell Signaling Technology™ and directed against phosphorylated forms of JNK and unphosphorylated and phosphorylated forms of MEK and ERK. Non-phospho-antibodies detect total levels of endogenous unphosphorylated MEK and ERK. Phospho-antibodies recognize phosphorylated MAP kinases. pMEK antibody signals were normalized against total MEK, while pERK, and pJNK antibody signals were normalized against total ERK. We tested for cross-reactivity with guinea pig and found bands specifically labeled for the unphosphorylated and phosphorylated forms of MEK, the unphosphorylated and phosphorylated forms of ERK, and pJNK. We could not test for p38 activation due to a lack of a suitable cross-reactive antibody. After blocking, membranes were incubated with primary antibodies in wash buffer (pH = 7.5; TBS with 0.1% Tween-20) on an orbital shaker over night at +4°C and then washed with wash buffer. After washing, membranes were incubated with enzyme-conjugated secondary antibodies (goat-anti-rabbit IgG) for 1 h at room temperature. After incubation, the same wash process was carried out. Substrate solution (SuperSignal^® ^West Femto; Pierce) was added to the blots and incubated for 5 min. Luminescence signals were detected using a Kodak image analyzer and membrane images were densitometrically analyzed using the TotalLab software (Nonlinear Dynamics Ltd, Newcastle upon Tyne, United Kingdom).

Expression of phosphorylated MEK (pMEK) was normalized to total MEK, phosphorylated ERK (pERK), and phosphorylated JNK (pJNK) were normalized to total ERK expression in fetal lungs from the same experimental conditions.

### Statistics

Values are presented as mean ± standard deviation (SD). Statistical analysis was carried out with one-way analysis of variance (ANOVA) with Tukey's test *post hoc*. Differences were considered statistically significant when *P *< 0.05 was reached.

## Results

### pERK, pMEK, and pJNK expression

**Figure 1:**
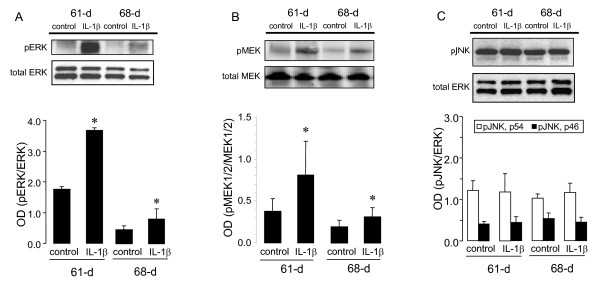
**A-C. **pERK (**A**), pMEK (**B**), and pJNK (**C**) expression in 61 and 68D gestation fetal guinea pig lungs with and without maternal IL-1β pretreatment. pERK and pJNK were normalized to total ERK. pMEK was normalized to total MEK1/2. Representative western blots for pERK, pMEK, and pJNK, as well as total ERK and MEK1/2 are shown. *P < 0.05 compared to age-matched control (ANOVA with Tukey's test *post hoc*).

pERK was studied in 61 and 68D gestation fetal lungs with and without maternal IL-1β pretreatment. IL-1β strongly increased pERK expression at 61D gestation, while having a much smaller effect at 68D gestation (Fig. 1A).

pMEK was studied in 61 and 68D gestation fetal lungs with and without maternal IL-1β pretreatment. IL-1β, similarly to pERK, increased pMEK expression at 61D gestation, while having a smaller effect at 68D gestation (Fig. 1B).

pJNK (p54 and p46) was studied in 61 and 68D gestation fetal lungs with and without maternal IL-1β pretreatment. IL-1β did not affect pJNK expression in either group (Fig. 1C).

### ERK inhibition

**Figure 2:**
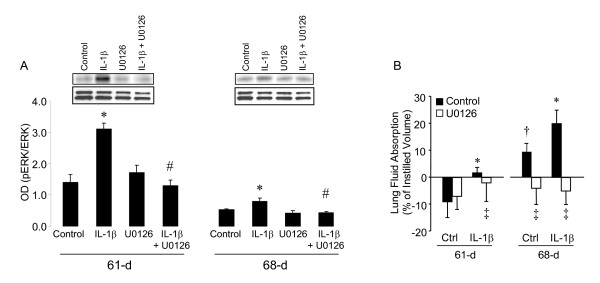
**AB**. pERK expression (**A**) in 61 and 68D gestation fetal guinea pig lungs with and without maternal IL-1β pretreatment and with and without instillation of U0126 (10^-6 ^M). pERK was normalized to total ERK. Representative western blots for pERK and total ERK are shown. *P < 0.05 compared to age-matched control; #*P *< 0.05 compared to age-matched IL-1β (ANOVA with Tukey's test *post hoc*). Lung fluid absorption (**B**) in 61 and 68D gestation fetuses with and without maternal IL-1β pretreatment with and without instillation of U0126 (10^-6 ^M). *P < 0.05 compared to age-matched control; †*P *< 0.05 compared to 61D control; ‡*P *< 0.05 compared to group control (ANOVA with Tukey's test *post hoc*).

We tested if MEK inhibition by its inhibitor U0126 affected pERK expression in IL-1β-pretreated fetal lungs. U0126 was instilled with the 5% albumin solution and lung tissue was assayed for pERK expression. IL-1β-induced/stimulated pERK expression was attenuated in both 61 and 68D gestation fetal lungs (Fig. 2A).

### ERK inhibition and lung fluid absorption

Lung fluid absorption was studied in fetal guinea pigs (61 and 68D gestation) after IL-1β pretreatment. Control 61D gestation fetal lungs were still secreting fluid and control 68D gestation fetal lungs absorbed lung fluid (Fig. 2B). Maternal IL-1β injections induced lung fluid absorption at 61D gestation and stimulated lung fluid absorption at 68D gestation (Fig. 2B). Co-administration of the MEK inhibitor U0126 to 61 and 68D gestation IL-1β-exposed fetuses attenuated IL-1β-induced/stimulated lung fluid absorption (Fig. 2B), but had little or no effect in 61D control fetuses.

### Cortisol synthesis inhibition, lung fluid absorption, and pERK expression

**Figure 3:**
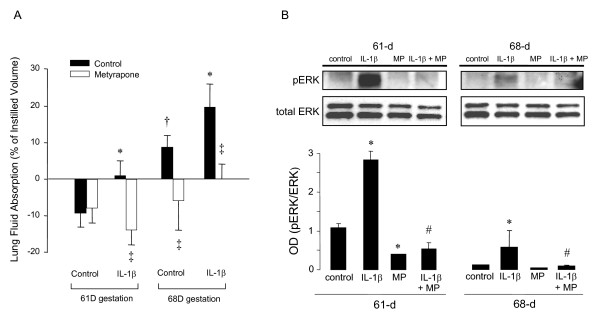
**AB. **Lung fluid absorption (**A**) in 61 and 68D gestation guinea pig fetuses with and without maternal IL-1β pretreatment with and without cortisol synthesis inhibition by MP.  *P < 0.05 compared to age-matched control; †*P *< 0.05 compared to 61D control; ‡*P *< 0.05 compared to group control (ANOVA with Tukey's test *post hoc*). pERK expression (**B**) in 61 and 68D gestation fetuses with and without maternal IL-1β pretreatment with and without cortisol synthesis inhibition by MP. Representative western blots for pERK and total ERK are shown. *P < 0.05 compared to age-matched control; #*P *< 0.05 compared to age-matched IL-1β (ANOVA with Tukey's test *post hoc*).

Lung fluid absorption and pERK expression were investigated in fetal guinea pigs (61 and 68D gestation) after IL-1β pretreatment with and without MP pretreatment. Control 61D gestation fetal lungs were not affected by MP pretreatment and in control 68D gestation fetal lungs MP pretreatment reversed lung fluid absorption to fluid secretion (Fig. 3A). IL-1β-induced lung fluid absorption at 61D gestation was also reversed to fluid secretion and IL-1β-stimulated lung fluid absorption at 68D gestation was completely inhibited by MP pretreatment (Fig. 3A). IL-1β-induced pERK expression at 61D gestation was also attenuated by MP pretreatment (Fig. 3B). MP pretreatment had less effect on 68D gestation pERK expression, although the IL-1β-stimulated pERK expression was attenuated (Fig. 3B).

## Discussion

This study expands on two earlier investigations from our laboratory [3,16] and investigates parts of the intracellular signaling machinery responsible for transducing the signal from IL-1β to an induced or stimulated fetal lung fluid absorption. The novel finding in this study was that MAP kinase activation followed maternal IL-1β exposure and elevated plasma cortisol concentrations and seemed to be at least partly responsible for the induced and stimulated fluid absorption rates at 61 and 68D gestation, respectively. Guinea pig lungs convert from fluid secretion to fluid absorption 3–5 days before birth [4,5]. The successful transition from fluid secretion to absorption in the lung is directly related to infant breathing and postnatal lung function. Several recent studies have suggested a novel role for IL-1 in lung maturation [14-16], where IL-1β may accelerate lung maturation in guinea pigs by accelerating the epithelial conversion to lung fluid absorption during gestation [3,16]. It has been demonstrated in several studies [2,9,10,20] that lung fluid is reabsorbed secondary to Na absorption. The molecular mechanism for this has been suggested to be the epithelial Na channel (ENaC) and this channel is sensitive to amiloride inhibition [2,6,9,10,21].

MAP kinases such as ERK and JNK have previously been demonstrated to be activated by cytokines and stress responses [22-25]. Although the activation of the MEK/ERK pathway and its downstream transcription factors are the best characterized [26], this signaling cascade has also been reported in regulation of multiple post-transcriptional mechanisms related to the translational machinery. This occurs primarily via regulation of the eukaryotic initiation factor 4E (eIF4E) and the p70^s6K ^[27-29]. In multiple investigations, it has been demonstrated in adult rats that dopamine and isoproterenol as well as growth factors can upregulate Na,K-ATPase expression via activating the MEK/ERK MAP kinase pathway [17,30-33]. Mostly, ERK activation led to increased expression and function of basolateral Na,K-ATPases [17,30,33] and some studies have suggested that activation of mTOR and p70^s6K ^were important downstream in stimulating increased Na,K-ATPase activity and expression [34]. However to our knowledge no study has investigated the effect of MAP kinase activation on ENaC function and lung fluid absorption in fetal lungs. In preliminary unpublished results from our laboratory activation of the MAP kinase pathway did not change lung ENaC expression. From these results, we cannot tell if ENaC activity was altered by MAP kinase activation. However, our data supports an effect on transepithelial Na^+ ^transport that may relate to MAP kinase stimulation of the Na,K-ATPase, as demonstrated earlier [17,30-34], as well as resulting in a secondary ENaC activation. Thus, we hypothesized that maternal IL-1β pretreatment and its effect on induction of cortisol synthesis and release is mediated in part by activation of ERK and JNK pathways in developing fetal guinea pig lungs. IL-1β pretreatment increased pMEK and pERK expression in fetal guinea pig lungs, but did not affect pJNK expression. This increased pERK expression was attenuated by intratracheal administration of the MEK inhibitor, U0126. Also, simultaneous administration of U0126 attenuated the IL-1β-induced/stimulated lung fluid absorption in the 61 and 68D gestation fetal lungs, suggesting that the ERK pathway was involved in IL-1β increased lung fluid absorption.

It has been reported earlier that IL-1β acts on the hypothalamus to stimulate release of hypothalamic corticotrophin releasing factor (CRF) and thus activate the hypothalamus-pituitary-adrenal gland axis with release of adrenocorticotropic hormone (ACTH) and plasma cortisol [16,35]. Therefore, we hypothesized that IL-1β increased lung fluid absorption at least partly via the hypothalamus-pituitary-adrenal gland axis and plasma cortisol synthesis and release in maternal animals and/or fetuses. As demonstrated earlier [16], MP pretreatment attenuated the IL-1β-induced/stimulated lung fluid absorption in fetal guinea pig lungs. Moreover, MP pretreatment completely inhibited pERK expression normally observed after maternal IL-1β pretreatment. This is a significant observation since there has been little prior evidence that plasma cortisol may affect MAP kinase signaling pathways.

The involvement of the ERK pathway in fluid clearance late in gestation may not be significant, as the 68D gestation lungs demonstrated a smaller ERK activation, but still a statistical increase in lung fluid absorption. Albeit the reasons for this may be plentiful, including little or no involvement of ERK at this gestation age, there were larger variations in the rates of both baseline lung fluid absorption and IL-1β-stimulated lung fluid absorption at 68D gestation than at 61D gestation. This may have resulted from release of variable endogenous plasma stress hormones, as these rises late during gestation as term approaches. It is inherently difficult to obtain homogenous guinea pigs at this gestation age and consequently the variation may be expected to be greater. All of this potentially leading to a false positive or negative result in signaling pathway function, since the same factors we are introducing via the IL-1β injections, ACTH and cortisol, are also being endogenously released at this gestation stage [16].

In conclusion, the results from this study suggest that MAP kinase activation after maternal IL-1β exposure with its resulting elevation of plasma cortisol concentrations may have been involved in controlling the extent of induction of fetal lung fluid absorption. This also suggests an intracellular control mechanism that might prove useful to pharmacologically induce in order to accelerate the conversion from lung fluid secretion to lung fluid absorption in the preterm lung in order to prevent respiratory syndrome development.

## References

[B1] Bland RD, Crystal RG, West JB (1991). Fetal lung liquid and its removal near birth. The lung: Scientific foundations.

[B2] Matthay MA, Folkesson HG, Clerici C (2002). Lung epithelial fluid transport and the resolution of pulmonary edema. Physiol Rev.

[B3] Nair PK, Li T, Bhattacharjee R, Ye X, Folkesson HG (2005). Oxytocin-induced labor augments IL-1β-stimulated lung fluid absorption in fetal guinea pig lungs. Am J Physiol Lung Cell Mol Physiol.

[B4] Finley N, Norlin A, Baines DL, Folkesson HG (1998). Alveolar epithelial fluid clearance is mediated by endogenous catecholamines at birth in guinea pigs. J Clin Invest.

[B5] Norlin A, Folkesson HG (2001). Alveolar fluid clearance in late gestational guinea pigs after induction of labor: mechanisms and regulation. Am J Physiol Lung Cell Mol Physiol.

[B6] Folkesson HG, Matthay MA, Chapin CJ, Porta NFM, Kitterman JA (2002). Pre- and postnatal lung development, maturation, and plasticity: Distal air space epithelial fluid clearance in near-term rat fetuses is fast and requires endogenous catecholamines. Am J Physiol Lung Cell Mol Physiol.

[B7] Nici L, Dowin R, Gilmore-Hebert M, Jamieson JD, Ingbar DH (1991). Upregulation of rat lung Na-K-ATPase during hyperoxic injury. Am J Physiol Lung Cell Mol Physiol.

[B8] Hummler E, Barker P, Gatzy J, Beermann F, Verdumo C, Schmidt A, Boucher R, Rossier BC (1996). Early death due to defective neonatal lung liquid clearance in α ENaC-deficient mice. Nat Genet.

[B9] Folkesson HG, Norlin A, Baines DL (1998). Salt and water transport across the alveolar epithelium in the developing lung: Correlations between function and recent molecular biology advances (Review). Int J Mol Med.

[B10] Li T, Folkesson HG (2006). RNA interference for α-ENaC inhibits rat lung fluid absorption *in vivo*. Am J Physiol Lung Cell Mol Physiol.

[B11] Romero R, Wu YK, Brody DT, Oyarzun E, Duff GW, Durum SK (1989). Human decidua: a source of interleukin-1. Obstet Gynecol.

[B12] Taniguchi T, Matsuzaki N, Kameda T, Shimoya K, Jo T, Saji F, Tanizawa O (1991). The enhanced production of placental interleukin-1 during labor and intrauterine infection. Am J Obstet Gynecol.

[B13] Barker PM, Gowen CW, Lawson EE, Knowles MR (1997). Decreased sodium ion absorption across nasal epithelium of very premature infants with respiratory distress syndrome. J Pediatr.

[B14] Bry K, Lappalainen U, Hallman M (1997). Intraamniotic interleukin-1 accelerates surfactant protein synthesis in fetal rabbits and improves lung stability after premature birth. J Clin Invest.

[B15] Willet KE, Kramer BW, Kallapur SG, Ikegami M, Newnham JP, Moss TJ, Sly PD, Jobe AH (2002). Pre- and postnatal lung development, maturation, and plasticity: Intra-amniotic injection of IL-1 induces inflammation and maturation in fetal sheep lung. Am J Physiol Lung Cell Mol Physiol.

[B16] Ye X, Acharya R, Herbert JB, Hamilton SE, Folkesson HG (2004). IL-1β stimulates alveolar fluid clearance in fetal guinea pig lungs via the hypothalamus-pituitary-adrenal gland axis. Am J Physiol Lung Cell Mol Physiol.

[B17] Pesce L, Guerrero C, Comellas A, Ridge KM, Sznajder JI (2000). β-Agonists regulate Na,K-ATPase via novel MAPK/ERK and rapamycin-sensitive pathways. FEBS Lett.

[B18] Norlin A, Baines DL, Folkesson HG (1999). Role of endogenous cortisol in basal liquid clearance from distal air spaces in adult guinea-pigs. J Physiol (London).

[B19] Lowry OH, Rosebrough NJ, Farr A, Randall RJ (1951). Protein measurement with the Folin phenol reagent. J Biol Chem.

[B20] Berthiaume Y, Staub NC, Matthay MA (1987). Beta-adrenergic agonists increase lung liquid clearance in anesthetized sheep. J Clin Invest.

[B21] Alvarez-de-la-Rosa M, Rebollo FJ, Codoceo R, Gonzalez A (2000). Maternal serum interleukin 1, 2, 6, 8 and interleukin-2 receptor levels in preterm labor and delivery. Eur J Obstet Gynecol Reprod Biol.

[B22] Lin F-S, Lin C-C, Chien C-C, Luo S-F, Yang C-M (2005). Involvement of p42/p44 MAPK, JNK, and NF-κB in IL-1β-induced ICAM-1 expression in human pulmonary epithelial cells. J Cell Physiol.

[B23] Wang Q, Downey GP, Choi C, Kapus A, McCulloch CA (2003). IL-1 induced release of Ca^2+ ^from internal stores is dependent on cell-matrix interactions and regulates ERK activation. FASEB J.

[B24] Lo YY, Luo L, McCulloch CA, Cruz TF (1998). Requirements of focal adhesions and calcium fluxes for interleukin-1-induced ERK kinase activation and c-fos expression in fibroblasts. J Biol Chem.

[B25] Reunanen N, Westermarck J, Hakkinen L, Holmstrom TH, Elo I, Eriksson JE, Kahari VM (1998). Enhancement of fibroblast collagenase (matrix metalloproteinase-1) gene expression by ceramide is mediated by extracellular signal-regulated and stress-activated protein kinase pathways. J Biol Chem.

[B26] Wasylyk B, Hagman J, Gutierrez-Hartmann A (1998). Ets transcription factors: nuclear effectors of the Ras-MAP-kinase signaling pathway. Trends Biochem Sci.

[B27] Rhoads RE (1999). Signal transduction pathways that regulate eukaryotic protein synthesis. J Biol Chem.

[B28] Waskiewicz AJ, Flynn A, Proud CG, Cooper JA (1997). Mitogen-activated protein kinases activate the serine/threonine kinases Mnk1 and Mnk2. Embo J.

[B29] Weng QP, Kozlowski M, Belham C, Zhang A, Comb MJ, Avruch J (1998). Regulation of the p70 S6 kinase by phosphorylation *in vivo*. Analysis using site-specific anti-phosphopeptide antibodies. J Biol Chem.

[B30] Guerrero C, Lecuona E, Pesce L, Ridge KM, Sznajder JI (2001). Dopamine regulates Na-K-ATPase in alveolar epithelial cells via MAPK-ERK-dependent mechanisms. Am J Physiol Lung Cell Mol Physiol.

[B31] Guerrero C, Pesce L, Lecuona E, Ridge KM, Sznajder JI (2002). Dopamine activates ERKs in alveolar epithelial cells via Ras-PKC-dependent and Grb2/Sos-independent mechanisms. Am J Physiol Lung Cell Mol Physiol.

[B32] Upadhyay D, Correa-Meyer E, Sznajder JI, Kamp DW (2003). FGF-10 prevents mechanical stretch-induced alveolar epithelial cell DNA damage via MAPK activation. Am J Physiol Lung Cell Mol Physiol.

[B33] Upadhyay D, Lecuona E, Comellas A, Kamp DW, Sznajder JI (2003). Fibroblast growth factor-10 upregulates Na,K-ATPase via the MAPK pathway. FEBS Lett.

[B34] Pesce L, Comellas A, Sznajder JI (2003). β-adrenergic agonists regulate Na-K-ATPase via p70^S6k^. Am J Physiol Lung Cell Mol Physiol.

[B35] Sapolsky R, Rivier C, Yamamoto G, Plotsky P, Vale W (1987). Interleukin-1 stimulates the secretion of hypothalamic corticotropin-releasing factor. Science.

